# Validity of the Mediterranean Diet and Culinary Index (MediCul) for Online Assessment of Adherence to the ‘Traditional’ Diet and Aspects of Cuisine in Older Adults

**DOI:** 10.3390/nu10121913

**Published:** 2018-12-04

**Authors:** Sue Radd-Vagenas, Maria A. Fiatarone Singh, Kenneth Daniel, Yian Noble, Nidhi Jain, Fiona O’Leary, Yorgi Mavros, Megan Heffernan, Jacinda Meiklejohn, Yareni Guerrero, Tiffany Chau, Perminder S. Sachdev, Henry Brodaty, Victoria M. Flood

**Affiliations:** 1 The University of Sydney, Physical Activity, Lifestyle, Ageing and Wellbeing Research Group, Faculty of Health Sciences, Lidcombe, NSW 2141, Australia; sue.radd-vagenas@sydney.edu.au (S.R.-V.); maria.fiataronesingh@sydney.edu.au (M.A.F.S.); kenneth.daniel@sydney.edu.au (K.D.); yian.noble@sydney.edu.au (Y.N.); nidhi.jain@sydney.edu.au (N.J.); yorgi.mavros@sydney.edu.au (Y.M.); jacinda.meiklejohn@sydney.edu.au (J.M.); yareni.guerrero@sydney.edu.au (Y.G.); 2 The University of Sydney, Sydney Medical School, Camperdown, NSW 2006, Australia and Hebrew SeniorLife and Jean Mayer USDA Human Nutrition Research Center on Aging at Tufts University, Boston, MA 02111, USA; 3 The University of Sydney, Nutrition and Dietetics Group, School of Life and Environmental Science, Faculty of Science and The Charles Perkins Centre, Camperdown, NSW 2006, Australia; fiona.oleary@sydney.edu.au; 4 Centre for Healthy Brain Ageing (CHeBA), School of Psychiatry, University of New South Wales, Randwick, NSW 2031, Australia; meganh@unsw.edu.au (M.H.); tiffany.chau@unsw.edu.au (T.C.); p.sachdev@unsw.edu.au (P.S.S.); h.brodaty@unsw.edu.au (H.B.); 5 Dementia Centre for Research Collaboration, University of New South Wales, Sydney, NSW 2052, Australia; 6 Western Sydney Local Health District, Westmead Hospital, Westmead, NSW 2145, Australia

**Keywords:** validity, reliability, repeatability, dietary assessment, index tool, score, Mediterranean diet, Mediterranean dietary pattern, traditional

## Abstract

The Mediterranean diet is associated with multiple health benefits. Yet, no tool has been specifically developed to assess adherence to the ‘traditional’ Mediterranean diet and cuisine within a Western cohort, and validated for online use. We tested the reliability and validity of online administration of the Mediterranean Diet and Culinary Index (MediCul) among middle-aged and older adults. Participants were recruited in January–March 2017 from the 45 and Up Study, completing MediCul twice. Test-retest reliability was assessed using the paired *t*-test, intra-class correlation coefficient (ICC) and Bland-Altman plot. Validity was tested against a three-day food record (FR)-derived MediCul score using Bland-Altman and nutrient trends across the MediCul score tertiles. Participants (*n* = 84; 60% female; 65.4 years (SD = 5.9)), were overweight (BMI 26.1; SD = 4.0) with 1.7 (SD = 1.5) chronic illnesses/conditions. Sequential MediCul tool scores were 56.1/100.0 and 56.8/100.0, respectively (*t* = −1.019; *p* = 0.311). Reliability via ICC (ICC = 0.86, 95% CI: 0.789, 0.910, *p* < 0.0001) and Bland-Altman was good. In Bland-Altman validity analyses, the tool over-reported FR MediCul score by 5.6 points with no systematic bias ((y = 8.7 − 0.06*x) (95% CI: −0.278, 0.158, *p* = 0.584)). Nutrient trends were identified for MediCul consistent with expected Mediterranean patterns. Online MediCul administration demonstrated good reliability and moderate validity for assessing adherence to a ‘traditional’ Mediterranean pattern among older Australians.

## 1. Introduction

The Mediterranean diet is a focus of research interest worldwide, as it has been associated with multiple health benefits. These include a reduced risk of premature death, cardiovascular disease (CVD), metabolic syndrome, cancer, liver disease, type 2 diabetes, depression, cognitive decline with ageing, mild cognitive impairment (MCI) and dementia, in particular Alzheimer’s disease [1,2,3,4,5,6,7,8,9,10,11].

However, there has been inconsistency in the literature with regards to the definition used for the Mediterranean diet [12,13]. Also, variable or inadequately reported Mediterranean interventions have been used in clinical trials, which may not represent the ‘traditional’ diet [7]. The ‘traditional’ Mediterranean diet has been described as the dietary pattern existing in Greece and Southern Italy during the late 1950s and early 1960s [13]. Briefly, it pertains to a more frugal, plant-based pattern with higher intakes of unrefined foods, such as grains, vegetables, fruits, legumes and extra virgin olive oil, and lower levels of exposure to meats, dairy and modern discretionary foods.

While the benefits ascribed to a Mediterranean diet parallel those of other plant-based diets, such as vegetarian diets and the Dietary Approaches to Stop Hypertension (DASH) diet [14,15], the ‘traditional’ Mediterranean diet also includes some unique cuisine aspects. For example, low advanced glycation end products (AGEs) cooking methods [16], such as boiling and stewing, and the use of acidic ingredients in cooking, such as lemon or vinegar; particular food combinations, e.g., onions sautéed in olive oil with tomato as the basis for many cooked dishes; regular inclusion of fermented foods, e.g., olives, feta cheese; limited snacking between meals; and water as the main beverage. Such elements may contribute to increased antioxidant bioavailability, reduced chronic systemic inflammation and positively influence the microbiome, but they have been rarely captured in existing tools [13]. Hence, previous Mediterranean diet index tools may not have been sufficiently nuanced to assess adherence to the ‘traditional’ Mediterranean diet and cuisine [17,18,19]. This may partly explain differences in reported health outcomes, associated with a Mediterranean diet score across various studies [12,20,21]. Some findings in the literature ascribed to the ‘traditional’ Mediterranean diet may simply represent increased adherence to healthier plant-based dietary patterns, which have also been associated with benefits, such as reduced risk of type 2 diabetes and mortality, outside the context of a particular diet [20,21].

Additional limitations of existing Mediterranean diet index tools, especially when used within a Western population, include the type and number of elements assessed, source of cut-off points used, score derivation methodology and lack of validation against a dietary reference method, which is not limited by the same recall biases [22]. For example, the number of components assessed in reported indexes has ranged from only six [23] to 28 in the MEDiterranean LIFEstyle index (MEDLIFE), which evaluates the Mediterranean diet and lifestyle more broadly [24]. Studies in Western countries have used sex-specific median values from their respective populations as cut-off points for the elements detailed in the first Mediterranean Diet Score (MDS) [11,25], originally developed for use in a Greek population. This is problematic, since the median intakes for certain foods, such as meat and dairy, are high in some Western cohorts [12]. Most Mediterranean diet adherence scores, including multiple variants of the MDS, the MedDietScore [26] and Mediterranean Style Dietary Pattern Score (MSDPS) [27], require derivation from a food frequency questionnaire (FFQ) rather than being calculated directly from responses to an administered index tool. Notable exceptions are MEDLIFE [24], the Mediterranean Diet Adherence Screener (MEDAS) [28] from the PREvencion conDIeta MEDiterranea (PREDIMED) study in Spain and the Mediterranean Eating Pattern for Americans (MEPA) screener adapted from MEDAS [29].

Only some Mediterranean diet index tools attempt to adjust for Western dietary habits or non-typical Mediterranean foods, such as discretionary foods [24,28,29,30,31], which were not part of the ‘traditional’ diet and may provide different health effects. Finally, in the era of e-Health, which opens the potential for wider tool administration including regional and remote areas, to the best of our knowledge, no Mediterranean diet index tool based on the ‘traditional’ diet and cuisine has been validated for online administration. Such a tool could assist with broad population-based and longitudinal studies.

We recently developed the Mediterranean Diet and Culinary Index (MediCul) tool, to overcome these common limitations. We have tested the performance of MediCul when administered in-person, to a cohort of older Australians with MCI [22]. As MediCul has the potential for wider use, the purpose of the present study was to test its reliability and validity when administered online to middle-aged and older people in the general population, living in the same Western country.

## 2. Materials and Methods

### 2.1. Participants

We aimed to recruit a cross-sectional sample of 100 [32,33] community-dwelling participants living in Sydney from January to March 2017, from the Sax Institute’s 45 and Up Study [34], as part of a validation study for various tools to be used in the *Maintain Your Brain* (MYB) trial administered within an online platform [35]. The 45 and Up Study is a large prospective cohort in New South Wales (NSW), Australia (*n* = 267,153) of participants recruited from the Department of Human Services (formerly Medicare) enrolment database representing diverse socio-demographic, geographic and cultural backgrounds, investigating healthy ageing [34]. Participants initially joined the study by completing the baseline questionnaire (between January 2006 and December 2009) and giving their consent for follow-up and linkage of their information to routine health databases.

Inclusion criteria for the MYB validity study were: (1) Previously enrolled in the 45 and Up Study and agreeable to further contact with email on record, (2) aged 55–75 years in 2016, (3) absence of dementia, Parkinson’s disease and multiple sclerosis, (4) home internet/computer access, (5) access to iPad or iPhone (for activity and diet logging), and (6) residing in postcode within 30 km radius of Lidcombe, NSW (site of clinic visit in Sydney).

Exclusion criteria were: (1) Unable to use a computer, (2) life threatening condition, (3) inability to write/converse in English, (4) serious psychiatric condition, on antipsychotics or suicidal ideation, and (5) unstable medical condition.

A potentially eligible subset of the 45 and Up Study cohort (*n* = 27,848) was identified by the Sax Institute, which administers the 45 and Up Study [34], based on above inclusion criteria except for home internet/computer access and access to iPad/iPhone, details which were unavailable to the Sax Institute. Based on an estimated recruitment rate of 30%, 300 invitations were initially sent by the Sax Institute to a random sample of participants. A further 303 invitations were sent later to achieve the target sample.

Participation in the MYB validity study was voluntary and electronic informed consent was obtained before starting any data collection. This was followed by full online screening for study eligibility, using both the inclusion and exclusion criteria. Ethics approval for MYB was obtained from the University of New South Wales Human Research Ethics Committee (#16252) and NSW Population and Health Services Ethics Committee (#2016/03/636). Ethical conduct for the 45 and Up Study was approved by the University of New South Wales Human Research Ethics Committee (HREC).

### 2.2. Assessments

The MYB validity study included four assessment time points relevant to the validation of the MediCul tool. MediCul was administered twice online utilizing SurveyMonkey^®^ (www.surveymonkey.com), at least one week apart (survey A and survey B), with an invitation for a clinic visit in between. The clinic visit was used to measure: (a) Weight in light clothing, without shoes, using calibrated digital scales (AND HW-100K and Avery Berkel HL122), (b) height using a wall-mounted Harpenden stadiometer (Holtain Limited, Crymmych, Pembrokeshire, UK), (c) waist circumference taken at the umbilicus with a flexible tape measure (Grafco^®^, Graham-Field, Model # 17-1340-2), and (d) resting heart rate, taken manually, after a five-minute seated resting period. All anthropometric measurements were made in triplicate with the mean being used. Body mass index (BMI) was calculated by dividing weight (kg) by height^2^ (m). Data relating to demographics, self-reported medical conditions, number of medications taken and supplement use were also collected as part of a suite of surveys during the first MediCul (survey A) administration. Composite medications were counted according to the number of active ingredients they contained. Prevalence of nutrition supplement use was based on any reported vitamin/mineral/botanical supplements, excluding probiotics. Vitamin B12 injections were counted as medication. At the end of the clinic visit, or via email if a clinic visit was not possible, participants were asked to keep a three-day food record (FR) as soon as they had completed MediCul survey B, selecting one weekend and two weekdays, which needed to be within a seven-day period, but did not need to be consecutive. Participants were instructed to use the ‘Research Food Diary’ app [36] as this may reduce participant and researcher burden. This app has previously been reported to have a high level of acceptance by adults compared to a 24-hour dietary recall [37] and mobile nutrition apps, in general, have been shown in healthy populations to be comparable to traditional dietary assessment methods [38]. The Research Food Diary app is based on Australian food composition data, and entries can be directly imported into FoodWorks 8 Professional Edition software: 8.0.3553 (Xyris Software Pty Ltd., Brisbane, Australia). We provided detailed downloadable instructions for the Research Food Diary app, including color screen shots. Some participants however, declined to use the app so a hard copy FR template was provided. The FR instructions provided to all participants advised them to log typical days (not to change dietary intake), record any recipes, enter foods as consumed throughout the day, and estimate serve sizes using household measures (spoons, cups) or weights (grams (g)), if preferred, using kitchen scales. Participants were advised to expect a call from a dietitian shortly after submitting their FR. A phone call was made to each participant within 24–48 hours of receiving their FR to query unusual quantities and confirm entries were complete using a checklist for potentially forgotten foods, based on principles from the ASA24 Automated Self-Administered 24-hour Dietary Assessment Tool [39,40].

Where MediCul survey A or survey B was not completed within one week of administration, an email reminder was sent with a direct link to prompt participants to complete their surveys. If either survey still remained incomplete 1–2 weeks later, a second email reminder was sent. If FRs were not submitted within one week, participants also received a reminder email. If FRs were not submitted within two weeks, telephone reminders were made.

### 2.3. The MediCul Tool

MediCul is a 50-item short dietary survey developed empirically to assess adherence to a ‘traditional’ Mediterranean dietary pattern and aspects of cuisine [13] within a Western population. It applies reverse scoring for high exposures to foods that were not part of the ‘traditional’ pattern, such as modern discretionary foods, which now account for approximately 35% of the total energy intake of Australians [41]. A score for the popular MEDAS screener can also be derived from MediCul (Supplementary Table S1).

MediCul is scored between 0 and 100, with a higher score representing greater adherence, and takes approximately 20 minutes to complete. Details about MediCul development, elements, cut-off points, scoring and rationale have previously been reported as Supplementary Materials in the British Journal of Nutrition and this tool is freely available for download and use in research and education [22].

For the present validity study, MediCul was modified for online administration using SurveyMonkey with drop-down response options, logic and data limits applied to facilitate correct dietary reporting without a dietitian being present. These backend adjustments were tested for accurate performance by five researchers.

### 2.4. Dietary Analysis

We used Excel (MS Office Professional Plus 2013) to derive scores for both the MediCul and MEDAS tools [22]. The FRs were coded and entered into FoodWorks 8 and we selected AusBrands 2015 and AusFoods 2015 data sources which map to the AUSNUT 2011–2013 Food Standards Australia New Zealand (FSANZ) nutrient database for analysis by S.R.-V. As the food groups within FoodWorks draw on the concept of the USDA Food Patterns Equivalents Database (FPED), which counts hot chips/fries/crisps and legumes in the vegetable group, we manually adjusted serves for the FoodWorks vegetable group to exclude these discretionary and legume foods. We also manually added some legume serves to the legume protein foods group, which were not counted by FoodWorks. We did not analyze the nutrient contribution from supplements as our focus was on validating a dietary pattern.

### 2.5. Statistical Analysis

Statistical analyses were conducted using the Statistical Package for Social Sciences (SPSS) for Windows version 24 (SPSS, Inc., Chicago, IL, USA). Histograms, skewness, kurtosis and minimum and maximum values were used to examine distributions for normality and identify potential data entry errors for index scores, nutrients and foods groups. Means and standard deviations (SD) were calculated and reported for normally distributed nutrients whereas medians and the interquartile range was reported for non-normally distributed nutrients.

The derived MEDAS score from survey A (*n* = 84) was compared to the baseline MEDAS score reported for participants in PREDIMED, being the largest randomized controlled trial (RCT) of a Mediterranean diet [42]. Further, we estimated the percent of our cohort that reached previously defined Mediterranean thresholds [22] for selected foods/’traditional’ aspects of cuisine, determined by their FRs.

Test-retest reliability for MediCul was assessed using the paired t-test and intra-class correlation coefficient (ICC), which was classified as poor (<0.40), fair to good (≥0.4 and <0.75), and very good (≥0.75) [43]. A Bland-Altman plot was also used to determine the level of agreement between survey A and survey B time points, since a high correlation does not necessarily mean good agreement [44]. Kappa was utilized to check percent agreement within the same category [32] for 17 major food groups within MediCul and values were interpreted using cut-offs proposed by Landis and Koch [45]. We used Spearman’s correlation coefficient to assess whether the lag time between survey A and survey B was correlated with the difference in MediCul scores at the two time points. We also investigated whether selected categorical (i.e., sex, supplement use, primary grocery shopper, primary cook) or continuous (i.e., age, BMI) variables might be driving the improved performance for survey B, using Chi-square, linear regression and paired *t*-tests, as appropriate.

Concurrent validity for MediCul was determined by examining the Bland-Altman plot of the difference in MediCul scores between the survey tool and the FR versus the mean of the scores from the two assessment methods, with limits of agreement (LOA) being ±2 standard deviations (SD) from the mean difference. Bias was assessed using linear regression analysis. A paired *t*-test was conducted for scores from the survey at time points A, B, and the mean of AB versus the FR, to check the mean differences and confidence intervals (CI) across these comparisons.

We also indirectly validated MediCul by examining whether expected (Mediterranean) nutrient trends from the FR were associated with tertiles of the MediCul score. For positively or negatively skewed nutrients, we conducted a log 10 transformation, then re-checked their distribution for normality to determine whether parametric or non-parametric statistical tests should be applied. Normally distributed and normalized nutrients were compared across tertiles of the MediCul score derived from both survey A and the FR, using one-way ANOVA, while non-normally distributed nutrients were analyzed using the Kruskal-Wallis test. Variances were checked for equality using the Levene’s test; the Bonferroni post hoc test was applied for equal variances and the Games-Howell for unequal variances. First (linear)- and second (quadratic)-order polynomial contrasts were applied to test for nutrient trends across tertiles, as well as the line of best fit for normally distributed/normalized nutrients. The Jonckheere trend test was used for non-normally distributed nutrients.

As the FR did not include details for three MediCul questions (i.e., growing of own vegetables, weekly main meals eaten alone and fasting frequency), the validation of MediCul was based on scoring out of 97 whereas reliability analysis was based on scoring out of 100.

## 3. Results

**Figure 1:**
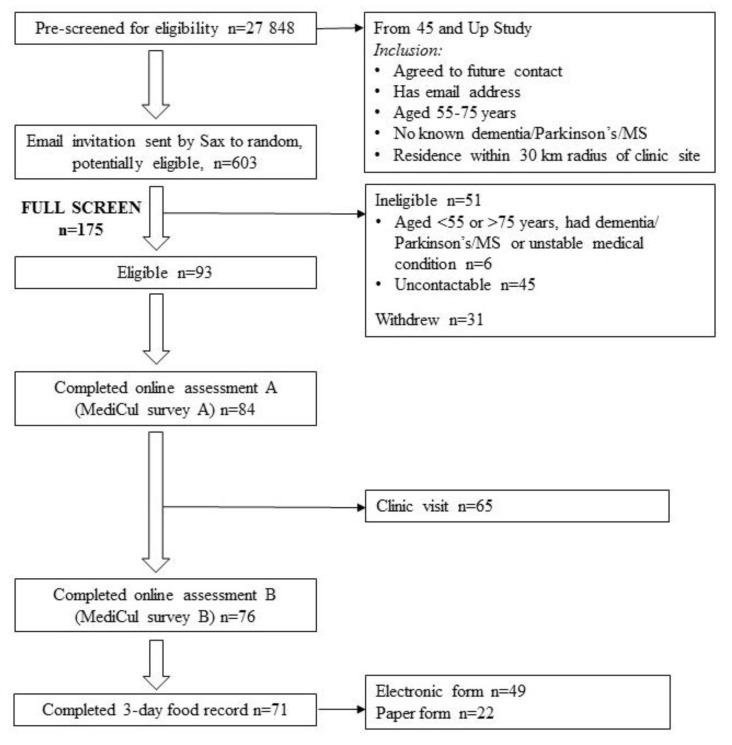
Flow chart of participants. Abbreviations: MediCul survey A, first administration of MediCul tool; MediCul survey B, second administration of MediCul tool; MS, multiple sclerosis; n, number of participants; Sax, the Sax Institute.

We received 175 responses to the 603 invitations sent by the Sax Institute to potentially eligible participants from the 45 and Up Study. Of these, 93 participants were eligible after full screening (Figure 1). A total of 84/93 (90%) completed the first administration of MediCul (survey A) and 65 participants elected to make a clinic visit for physical measurements. Seventy-six participants completed MediCul on two occasions. The final number included for reliability testing was 74, since two participants started their FR prior to completing MediCul survey B and were excluded. Validity testing was conducted with 71 participants who completed both survey A and the FR, with 69% of these using the app and submitting an electronic FR. Data collection for all participants was completed by mid-May 2017.

The majority of the recruited participants were female, and reported taking at least one nutritional supplement, with about one-half being the primary grocery shopper and primary cook at home (Table 1). Participants ranged in age from 56 to 76 years, were overweight on average (BMI = 26.1; SD = 4.0), with approximately one-half having a waist circumference associated with high chronic disease risk [46] (Table 1). They had 1.7 (SD = 1.5) chronic illnesses/conditions, on average, with the most prevalent being osteoarthritis, followed by hypercholesterolemia and hypertension. The median number of medications taken was 1.5 (IQR: 0.0, 3.0).

**Figure 2:**
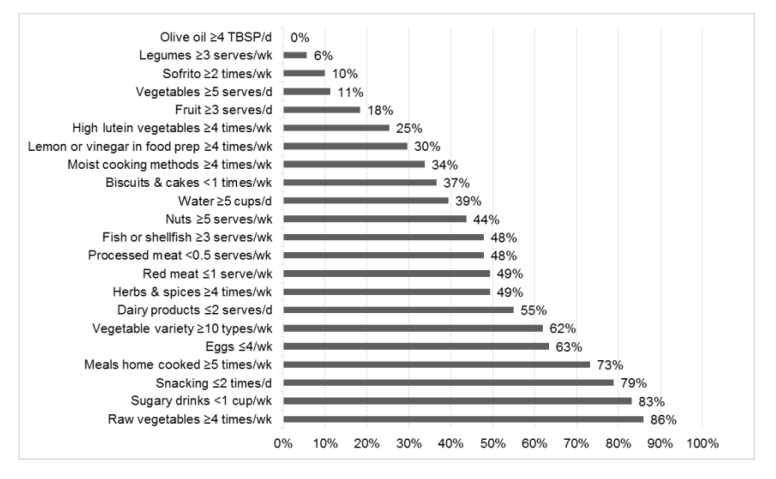
Percent of participants who reached Mediterranean diet thresholds according to three-day food records (*n* = 71). Thresholds defined by highest cut-offs used in MediCul tool for the relevant questions [22]. Abbreviations: *n*, number of participants; d, day; Prep, preparation; Sofrito, a sauce made with tomato, onion/garlic and olive oil, used as a base for savory dishes; TBSP, tablespoon; wk, week.

The mean MediCul score from survey A (*n* = 84) was 55.2/100.0 (SD = 11.6; range: 27.5, 75.5). The derived mean MEDAS score for the same time point was 6.5/14.0 (SD = 1.7; range: 2.0, 10.0), which was approximately two points lower than the baseline MEDAS score of 8.6 (SD = 2) [42] reported in the Spanish PREDIMED population, prior to intervention with a Mediterranean diet supplemented with extra virgin olive oil or nuts or a reduced fat control diet. However, it was one point higher than reported recently for a Western UK cohort [47]. Only 3/84 (4%) of our participants achieved a MEDAS score of ≥10, which has been classified as high adherence to a Mediterranean diet and associated with better health outcomes in PREDIMED (2, 48–50). All of these participants were in the highest tertile of the MediCul score from survey A (cut-off = 59.0 for tertile 3) when compared to nutrients from the FR. Few participants reached defined Mediterranean thresholds for selected foods/’traditional’ aspects of cuisine [22], especially for olive oil (0%), legumes (6%), sofrito, a sauce made with tomato, onion/garlic and olive oil, used as the base for many dishes (10%), vegetables (11%) and fruit (18%) (Figure 2). However, the majority reported cooking most of their main meals at home (73%), limiting snacking occasions (79%), limiting sugary drinks (83%) and consuming raw vegetables/salads most days of the week (86%). Almost half included an adequate intake of nuts (44%) and fish/shellfish (48%).

### 3.1. Reliability

There was a considerable lag time between MediCul survey A and survey B for some participants, so the time between surveys was non-normally distributed (median = 16 days; IQR: 13, 20). As we did not have an *a priori* criterion for the length of time between surveys, we did not exclude any participants for reliability testing based on the time between surveys.

MediCul B score was similar to MediCul A 56.8/100 and 56.1/100.0, respectively (*t* = −1.019; *p* = 0.311). The MediCul tool was highly reliable as assessed by ICC and 95% CI, (ICC = 0.86, 95% CI: 0.789, 0.910, *p* < 0.0001). Using the Bland-Altman test for repeated measures, the mean difference between the scores at the two time points was −0.69 with lower and upper LOA being −12.09 and 10.71, respectively. Seventy of the 74 participants (95%) fell within the LOA representing ±2 SD, with a reasonably even distribution of the mean difference in scores. There was no indication of bias according to the regression coefficient ((y= −2.95 + 0.04*x) (95% CI: −0.088, 0.168; *p* = 0.535)), further supporting the null hypothesis that the scores were equally variable at the two time points. The slightly higher mean score from survey B was unrelated to age, BMI, sex, supplement use, being the primary grocery shopper or primary cook. The lag time between repeated tool administration was unrelated to the difference between MediCul scores (Spearman’s *p* = −0.102; *p* = 0.385), suggesting that MediCul is a stable index for usual intakes across the time spans in this study.

Percent agreement within the same category of the 17 main MediCul food groups for survey A and survey B, determined by Kappa, was ‘almost perfect’ for coffee; ‘substantial’ for fruit, wholegrains, fish/shellfish, eggs, dairy products, takeaway and water; ‘moderate’ for olive oil, nuts, legumes, white meat preference, red/processed meat, sweets and sugary drinks and alcohol; and ‘fair’ for vegetables and cuisine (Supplementary Table S2). No groups were found to have poor agreement within the same category between the two time points of MediCul tool administration.

### 3.2. Validity

Analysis of the paired *t*-tests for MediCul scores from the survey administered at time points A, B and mean of AB versus the FR (*n* = 68) indicated a similar mean difference and 95% CI across the three comparisons, which were all statistically significant (*p* < 0.0001) (Table 2). We thus decided to proceed with survey A for the remaining validity testing, because this time point included most data points (*n* = 71) and represented first time MediCul use, which could be applied in future research.

**Figure 3:**
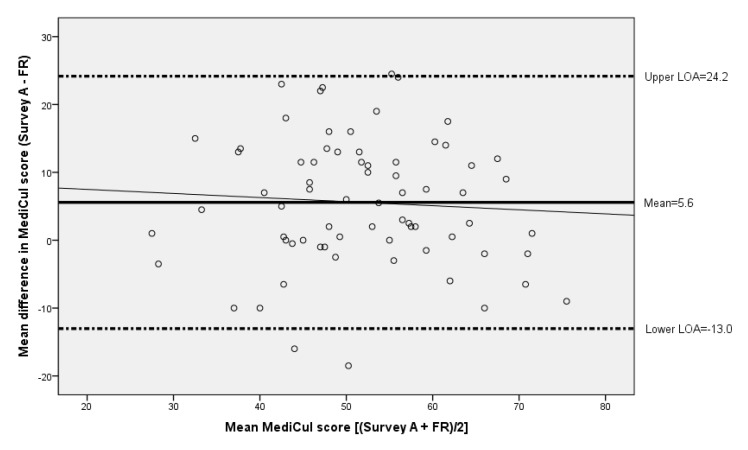
Bland-Altman plot of the difference in the Mediterranean Diet and Culinary Index (MediCul) score between survey A (first administration of MediCul tool) and three-day FR versus the mean MediCul score of the two methods (*n* = 71). The solid line indicates the mean difference, whereas the dotted lines are the limits of agreement representing ±2 SD from the mean difference within which 95% of the differences between the methods are expected to fall. The fitted regression line is ((y = 8.7−0.06*x) (95% CI: −0.278, 0.158, *p* = 0.584)), and indicates no significant linear trend. Abbreviations: FR, food record.

The Bland-Altman plot showed a mean difference of 5.6 between the MediCul score derived from survey A (54.6/97.0, SD = 11.2, range: 26.5, 73.5) and the FR (49.0/97.0, SD = 11.7, range: 25.0, 80.0), and the mean difference was lower among participants who provided a paper version of their FR compared to the electronic version (supplied through the app), although both were similarly checked by the dietitian (Table 2). The scores for 69/71 participants (97%) fell within or on the 95% LOA with lower and upper LOA being −13.0 and 24.2, respectively, with reasonably even distribution across mean scores. There was no significant linear trend for the fitted regression line ((y = 8.7 − 0.06*x) (95% CI: −0.278, 0.158, *p* = 0.584)), indicating no systematic bias between the two measurement methods (Figure 3).

We hypothesized that a higher intake of certain nutrients (determined by FR analysis) would be associated with the higher tool (survey A) and FR-derived MediCul scores, categorized as tertiles. This was true for: Polyunsaturated fatty acids (PUFA) as % fat, *n*-3 long chain (LC) PUFA (mg), eicosapentaenoic acid (EPA) (mg), docosahexaenoic acid (DHA) (mg), and ratio of unsaturated to saturated fatty acids (SFA), supporting increased adherence to a Mediterranean dietary pattern (Table 3). Additionally, we hypothesized that a lower intake of some other nutrients would be associated with the higher MediCul score tertiles from both sources. This was true for SFA as % fat, alcohol (g) and the sodium to potassium ratio, being consistent with the plant based, minimally processed, nature of the ‘traditional’ Mediterranean diet, which is proportionally lower in SFA and includes only a moderate amount of alcohol. For the sodium to potassium ratio, a significant difference (*p* = 0.004) was observed between MediCul score tertiles 1 and 3, derived from the FR.

For some nutrients, the expected relationship was observed only with the MediCul score tertiles from the FR e.g., PUFA (g), alpha linolenic acid (ALA) (mg), ratio of monounsaturated fatty acids (MUFA) to SFA, dietary fiber (g), vitamin E (mg), potassium (mg), magnesium (mg) and selenium (μg). For magnesium, not only was there a positive linear trend across tertiles of the MediCul score from the FR, but a significant difference was observed between tertiles 1 and 3 (*p* = 0.015) and tertiles 2 and 3 (*p* = 0.016). PUFA % fat, EPA and DHA are nutrient examples for which significant differences were found between tertiles 1 and 3 of the MediCul score, derived from both the tool and FR (Table 3).

In all cases linear trends were significant and there were no significant deviations from normality except for retinol equivalents (μg), beta-carotene (μg) and iron (mg), where a quadratic trend was a better fit for these nutrients in relation to the MediCul score tertiles from survey A.

There was no relationship between tertiles for either of the MediCul scores and intake of energy (kJ), protein (g), total fat (g), SFA (g), MUFA (g), cholesterol (mg), carbohydrate (g), total sugars (g) and folate (μg).

## 4. Discussion

Our study indicates that online administration of MediCul has good reliability and moderate validity, as an assessment of adherence to a ‘traditional’ Mediterranean dietary pattern and aspects of cuisine among the middle-aged and older individuals studied who live in a Western country. These findings are consistent with those reported for in-person administration among older individuals with MCI [22].

The participants included in our study were Australians aged 55–75 years, living in suburban Sydney. Only 4% had high adherence to a Mediterranean diet based on their derived MEDAS scores. Higher MEDAS scores have been related to positive health outcomes in PREDIMED [2,48,49,50]. Similarly, a very small proportion reached thresholds for ‘traditional’ Mediterranean foods or exposures. For example, 11% reported adequate vegetable intake, 6% had adequate legumes, and 49% limited their exposure to red meat, in accordance with ‘traditional’ levels. These participants represented a fairly homogenous group in regards to their dietary intakes, with few people achieving a high MediCul score. Such a cohort may be at higher chronic disease risk. Education on the Mediterranean diet, which is already recommended by dietary guidelines, may be prudent. Targets for ‘traditional’ Mediterranean foods and cuisine aspects can be viewed in previously published Supplementary Materials that describe cut-off points used in the MediCul tool [22]. For example, four tablespoons of extra virgin olive oil per day; one serve (30 g) of nuts, five or more days per week; inclusion of dark green leafy vegetables (e.g., spinach, kale, silverbeet), four or more times per week.

Concurrent validity of MediCul has therefore been demonstrated within a cohort that is almost entirely Western in their eating habits. Further research is required to validate MediCul use among other population groups, including those with broader dietary habits (particularly at the high end), and in relation to biomarkers of dietary intake. The MYB trial, a three-year randomized controlled trial of a fully online multi-modal intervention targeting modifiable dementia risk factors in 6236 participants, will utilize MediCul at baseline and annual follow up. This will generate data on change in MediCul score over time and its association with chronic disease risk factors, which will provide external validity, since clinical end points were not available from this validation study [35]. Given the good reliability of the MediCul tool shown here, we anticipate that changes in the index score, in the context of the MYB trial and similar dietary interventions, would represent true dietary change, and could potentially serve as a marker of positive health outcomes related to the ‘traditional’ diet and cuisine.

### Strengths and Limitations

The MediCul tool assesses the overall dietary pattern within a Western context, capturing the multi-dimensional nature of individual diets. With 50 items being assessed, it provides a comprehensive score for adherence to a ‘traditional’ Mediterranean dietary pattern and aspects of cuisine. Many of these elements are lacking from most other index tools. MediCul also enables derivation of a MEDAS score for comparison with studies that have used this short screener. Importantly, it is relatively simple to use and compute its scoring and MediCul can be administered online and in-person [22], increasing its potential utility, feasibility in large cohorts and cost-effectiveness.

Our study has some limitations. Data are currently lacking to support external validity for MediCul, which is of clinical relevance. Generalizability may also have been tempered by selection bias as our participants came from the 45 and Up Study, thus representing individuals who are better educated, more health aware and who may respond differently to dietary surveys compared to non-volunteers [32]. Our participants had less obesity and diabetes compared to the general Australian population for a similar age group [51,52]. MediCul was also administered within a suite of surveys, which may have caused fatigue, impacting on responses and scores. This was unavoidable as we wished to simulate the online testing experience of the subsequent MYB trial. Our testing, nevertheless, confirmed good reliability and moderate validity. It is also well known that food records may under-report intake or include atypical days [53] and there were some limitations specifically related to use of the Research Food Diary app. For example, certain recipes entered into the app by participants included some scanned foods that did not exist in the FoodWorks data source available to us, and needed to be re-entered by the dietitian, which was done by selecting nutritionally similar foods. If participants selected composite dishes within the app or reported a composite meal in a paper FR (e.g., stir fry chicken and vegetables), the dietitian checked all entries similarly during the phone call by probing for potentially missed foods and ingredients, such as oil. While the MediCul tool performed well overall, it performed better against a traditional (paper) FR, as compared to an electronic FR (using an app). However, it was not our intention to compare methods of keeping an FR, and we therefore did not exclude any participants who provided a paper FR as we did not have an *a priori* criterion to do so.

In some participants, the approximately 20 point difference in MediCul score between the new tool and the FR, could be clinically meaningful. However, it has been suggested that a certain degree of disagreement is to be expected when instruments assessing usual diet are validated against food records, due to within-person variations that typically occur over the shorter reference period of an FR [32]. Also, the lower and upper LOA (13% and 25%, respectively) were similar to findings for MediCul from our validity study among individuals with MCI [22] and well within the 50% and 200% LOA suggested by Ambrosini et al. [54] to classify agreement between an FFQ and food record as being acceptable. Furthermore, the new MediCul tool appears to be closer to the reference method, as compared to MEDAS. MEDAS under- and overestimated scores by 43% and 53%, respectively, when validated in a Spanish population against an FFQ [28], or by 16% and 36% when validated within a UK cohort against an FR [47].

## 5. Conclusions

MediCul is a highly reliable and moderately valid Mediterranean diet index tool for online administration among health aware, better educated, middle-aged and older individuals living in a Western country, which can be used to assess adherence to a ‘traditional’ dietary pattern and aspects of cuisine relevant to many important health outcomes.

## Figures and Tables

**Table 1:** Participant characteristics at baseline (*n* = 84).

Descriptive Statistics (Mean (SD) or Proportion)	
Age (years)	65.4 (5.9)
Females (%) BMI * (kg/m^2^)	60 26.1 (4.0)
<18.5 (%)	1.5
18.5 to 24.9 (%)	35.4
25.0 to 29.9 (%)	47.7
≥30 (%)	15.4
Waist circumference *—females (cm)	91.4 (12.4)
≥88 cm (%)	56.4
Waist circumference *—males (cm)	100.2 (8.2)
≥102 cm (%)	46.2
Resting heart rate * (beats per minute)	66 (8)
Primary cook at home (%)	49
Primary grocery shopper at home (%)	51
Supplement use (%)	63
Number of chronic illnesses/conditions	1.7 (1.5)
Osteoarthritis (%)	36
Hypercholesterolemia (%)	30
Hypertension (%)	24
Gastro-esophageal reflux disease (%)	16
Depression (%)	7
Diabetes (%)	4
Number of medications (median (IQR))	1.5 (0.0, 3.0)

Abbreviations: IQR, interquartile range representing the 25th and 75th percentiles; *n*, number of participants; SD, standard deviation; BMI, body mass index (calculated as weight in kilograms divided by height in meters squared). * measured at clinic visit (*n* = 65).

**Table 2:** Mean difference from paired samples t-test for the Mediterranean Diet and Culinary Index (MediCul) scores from surveys A, B, mean AB versus three-day food record (*n* = 68).

	Mean Difference	95% CI of the Difference	*p* Value
A versus FR *	5.32	3.03, 7.62	<0.0001
B versus FR	6.38	4.16, 8.59	<0.0001
Mean AB versus FR	5.85	3.70, 8.00	<0.0001

Abbreviations: A, first administration of MediCul; B, second administration of MediCul; mean AB, mean of A and B MediCul administrations; *n*, number of participants; FR, three-day food record; CI, confidence intervals. * When compared to Survey A with maximum data points (*n* = 71), participants using a paper FR (*n* = 22) had a mean difference of 2.6 points (95% CI: −2.36, 7.63; *t* = 1.10; *p* = 0.285), whereas those using the electronic FR (*n* = 49) had a mean difference of 6.9 points (95% CI: 4.50, 9.30; *t* = 5.77; *p* < 0.0001).

**Table 3:** Nutrient intakes compared with tertiles of the Mediterranean Diet and Culinary Index (MediCul) score from FR and survey A (*n* = 71) *.

Nutrients from Food Record	Source of the MediCul Score Tertile Cut-Off Points	Nutrient Intake for Tertile 1 of the MediCul Score		Nutrient Intake for Tertile 2 of the MediCul Score		Nutrient Intake for Tertile 3 of the MediCul Score		Comparison of Nutrient Intakes across Tertiles of the MediCul Score *p* Value	Test for Trend *p* Value, Direction ↑ ↓ †
		Mean/median	SD/IQR	Mean/median	SD/IQR	Mean/median	SD/IQR		
**Energy kJ/d**	**FR**	9206	2322	8330	2323	9054	2342	0.386	0.810
	**Survey**	9326	2450	8526	2124	8733	2435	0.468	0.380
**Protein g/d**	**FR**	97	25	89	22	101	20	0.190	0.548
	**Survey**	100	24	89	26	97	16	0.200	0.605
**Protein % energy**	**FR**	18	3	19	4	19	3	0.474	0.224
	**Survey**	19	3	18	4	20	4	0.208	0.301
**Fat g/d**	**FR**	87	31	77	24	93	33	0.189	0.491
	**Survey**	85	27	83	28	89	37	0.790	0.661
**Fat % energy**	**FR**	34	6	34	6	38	7	0.129	0.083
	**Survey**	34	5	36	6	37	8	0.242	0.097
**SFA g/d**	**FR**	32	11	26	11	29	13	0.266	0.412
	**Survey**	30	11	30	12	27	12	0.613	0.411
**SFA % energy**	**FR**	13	2	12	3	12	4	0.428	0.269
	**Survey**	12	2	13	3	11	3	0.191	0.588
**SFA % fat**	**FR**	41 ^a^	6	38	8	33	8	0.004	**0.001 ↓**
	**Survey**	39	7	39 ^b^	7	34	8	0.024	**0.030 ↓**
**PUFA g/d**	**FR**	12	9, 16	11 ^b^	9, 16	14	12, 22	0.017	**0.018 ↑**
	**Survey**	11	9, 15	12	10, 15	15	11, 22	0.146	0.087
**PUFA % fat**	**FR**	16 ^a^	5	18	5	21	7	0.021	**0.007 ↑**
	**Survey**	17 ^a^	5	17	5	21	7	0.026	**0.013 ↑**
**MUFA g/d**	**FR**	35	14	31	10	39	14	0.169	0.300
	**Survey**	35	11	33	12	37	16	0.594	0.559
**MUFA % fat**	**FR**	43	4	45	6	45	6	0.242	0.099
	**Survey**	45	5	43	5	45	7	0.453	0.721
***n*-3 LC PUFA mg/d**	**FR**	242 ^a^	125, 459	261 ^b^	97, 606	891	290, 1725	0.004	**0.006 ↑**
	**Survey**	241	112, 409	290	176, 670	858	291, 1583	0.035	**0.025 ↑**
**ALA mg/d**	**FR**	1499 ^a^	851, 1808	1445	966, 2005	1643	1437, 2757	0.027	**0.012 ↑**
	**Survey**	1539	908, 2112	1489	992, 1758	1591	1308, 2757	0.064	0.069
**EPA mg/d**	**FR**	70 ^a^	24, 138	72 ^b^	29, 200	372	78, 665	0.005	**0.006 ↑**
	**Survey**	66 ^a^	24, 117	74 ^b^	42, 243	275	81, 567	0.023	**0.009 ↑**
**DPA mg/d**	**FR**	69	47,129	55	29, 117	136	67, 312	0.054	0.083
	**Survey**	57	37, 129	70	49, 113	130	48, 308	0.141	0.123
**DHA mg/d**	**FR**	109 ^a^	43, 235	124 ^b^	30, 345	391	149, 751	0.005	**0.004 ↑**
	**Survey**	109 ^a^	42,197	149 ^b^	47, 328	409	162, 640	0.023	**0.009 ↑**
**MUFA:SFA ratio**	**FR**	1.1 ^a^	0.9, 1.2	1.1	0.9, 1.6	1.4	1.1, 1.7	0.010	**0.003 ↑**
	**Survey**	1.2	1.0, 1.4	1.1	0.9, 1.2	1.6	1.0, 1.7	0.053	0.082
**Unsaturated:SFA ratio**	**FR**	1.5 ^a^	0.3	1.8	0.6	2.1	0.7	0.002	**<0.001 ↑**
	**Survey**	1.7 ^a^	0.5	1.6 ^b^	0.5	2.1	0.8	0.007	**0.010 ↑**
**Cholesterol mg/d**	**FR**	333	155	264	105	322	130	0.153	0.761
	**Survey**	333	128	281	150	306	120	0.400	0.488
**Carbohydrate g/d**	**FR**	205	55	192	69	196	63	0.782	0.618
	**Survey**	208	63	197	58	187	66	0.500	0.241
**Carbohydrate % energy**	**FR**	37	7	38	6	35	7	0.448	0.461
	**Survey**	37	6	38	6	35	8	0.265	0.339
**Sugars g/d**	**FR**	96	34	98	41	94	39	0.948	0.851
	**Survey**	98	34	98	40	91	39	0.772	0.545
**Alcohol g/d**	**FR**	21 ^a^	8, 31	13	1, 26	7	1, 15	0.035	**0.010 ↓**
	**Survey**	27 ^a^	12, 34	8	2, 17	8	1, 15	0.014	**0.008 ↓**
**Water g/d**	**FR**	2878	517	3106	692	3089	827	0.447	0.293
	**Survey**	2995	466	2856	690	3245	837	0.148	0.229
**Dietary fiber g/d**	**FR**	27	8	30	10	33	9	0.064	**0.019 ↑**
	**Survey**	29	10	28	7	34	10	0.080	0.073
**Vitamin C mg/d**	**FR**	128	43, 151	120	82, 198	120	79, 166	0.156	0.084
	**Survey**	113	64, 166	128	73, 158	123	95, 202	0.190	0.092
**Vitamin E mg/d**	**FR**	12	11, 15	14	11, 18	17	12, 22	0.083	**0.042 ↑**
	**Survey**	13	12, 18	13	11, 15	18	11, 23	0.089	0.222
**Vitamin B12 μg/d**	**FR**	4.8	3.7, 5.9	4.2	3.1, 5.2	4.5	3.6, 6.3	0.215	0.793
	**Survey**	4.9	3.7, 6.5	4.4	3.5, 5.7	4.4	3.6, 5.0	0.524	0.472
**Folate μg/d** **‡**	**FR**	381	119	379	122	403	112	0.732	0.516
	**Survey**	389	123	363	108	414	119	0.321	0.484
**Retinol equivalents μg/d**	**FR**	1092	557	1193	568	1266	587	0.577	0.298
	**Survey**	1153	404	972 ^b^	557	1454	637	0.012	**0.016**
**Beta carotene μg/d**	**FR**	4567	3307	5619	3245	6047	3273	0.285	0.125
	**Survey**	5043	2477	4167 ^b^	3112	7196	3593	0.004	**0.013**
**Sodium mg/d**	**FR**	2330	753	2021	750	1920	688	0.141	0.058
	**Survey**	2298	836	2064	699	1901	652	0.190	0.071
**Potassium mg/d**	**FR**	3513	813	3690	1057	4105	945	0.096	**0.036 ↑**
	**Survey**	3691	860	3520	939	4124	1028	0.089	0.135
**Sodium:Potassium ratio**	**FR**	0.7 ^a^	0.3	0.6	0.2	0.5	0.2	0.006	**0.001 ↓**
	**Survey**	0.6	0.2	0.6	0.2	0.5	0.2	0.072	**0.031 ↓**
**Magnesium mg/d**	**FR**	390 ^a^	349, 500	361 ^b^	321, 466	470	440, 525	0.006	**0.005 ↑**
	**Survey**	418	333, 489	393	365, 469	466	365, 557	0.154	0.148
**Iron mg/d**	**FR**	12.1	3.3	11.7	3.4	13.2	3.5	0.288	0.254
	**Survey**	12.9	3.6	11.1	2.7	13.1	3.7	0.091	**0.030**
**Zinc mg/d**	**FR**	12.1	4.0	10.7	3.2	11.9	3.1	0.336	0.868
	**Survey**	11.8	4.2	11.1	3.1	11.8	3.1	0.721	0.960
**Selenium μg/d**	**FR**	89	70, 109	91	76, 105	103	92, 134	0.035	**0.017 ↑**
	**Survey**	96	81, 117	92	68, 109	96	83, 110	0.435	0.751

Abbreviations: *n*, number of participants; SD, standard deviation; IQR, interquartile range (representing the 25th and 75th percentiles); kJ, kilojoules; d, day; FR, food record; g, grams; SFA, saturated fatty acids; PUFA, polyunsaturated fatty acids; MUFA, monounsaturated fatty acids; *n*-3, omega 3; LC, long chain; mg, milligrams; ALA, alpha linolenic acid; EPA, eicosapentaenoic acid; DPA, docosapentaenoic acid; DHA, docosahexaenoic acid. * MediCul index score tertiles are derived from FR and survey A (first administration of MediCul). For survey A, the cut-off points for tertiles 2 and 3 were 50.0 and 59.0, respectively. Values are presented as mean (SD) for normally distributed data or median (IQR) for non-normally distributed data. These data were normalized by logarithmic transformation for use in ANOVA models with the exception of alcohol, selenium, EPA and DHA, which were unable to be normalized and therefore analyzed using the Kruskal-Wallis model. When ANOVA F ratio was significant, variances were checked for equality using the Levene’s test, and Bonferroni was applied for equal variances or Games-Howell post hoc *t* test for unequal variances. First (linear)- and second (quadratic)-order polynomial contrasts were applied to test for trends across tertiles, as well as the line of best fit for normally distributed/normalized nutrients. The Jonckheere trend test was used for non-normally distributed nutrients. In all cases linear trends were significant and there were no significant deviations from normality, except for retinol equivalents, beta carotene and iron when compared with tertiles from survey A, where the quadratic trend was positive and the most significant. Significant differences (*p* < 0.05) between tertiles denoted as: a = 1 vs. 3; b = 2 vs. 3. † Linear trend direction indicated as ↑ (increasing) or ↓ (decreasing). ‡ Naturally occurring food folates only.

## Supplementary Materials

The following are available online at http://www.mdpi.com/2072-6643/10/12/1913/s1, Table S1: How to derive a MEDAS score from MediCul and Table S2: Kappa statistics for percent agreement within the same category of 17 MediCul groups, between survey A and survey B.

## References

[1] Esposito K., Maiorino M.I., Ceriello A., Giugliano D. (2010). Prevention and control of type 2 diabetes by Mediterranean diet: A systematic review. Diabetes Res. Clin. Pract..

[2] Estruch R., Ros E., Salas-Salvado J., Covas M.I., Corella D., Aros F., Gomez-Gracia E., Ruiz-Gutierrez V., Fiol M., Lapetra J. (2018). Primary prevention of cardiovascular disease with a Mediterranean diet supplemented with extra-virgin olive oil or nuts. N. Engl. J. Med..

[3] Jacka F.N., O’Neil A., Opie R., Itsiopoulos C., Cotton S., Mohebbi M., Castle D., Dash S., Mihalopoulos C., Chatterton M.L. (2017). A randomised controlled trial of dietary improvement for adults with major depression (the ‘SMILES’ trial). BMC Med..

[4] Kastorini C.-M., Milionis H.J., Esposito K., Giugliano D., Goudevenos J.A., Panagiotakos D.B. (2011). The effect of Mediterranean diet on metabolic syndrome and its components: A meta-analysis of 50 studies and 534,906 individuals. J. Am. Coll. Cardiol..

[5] Parletta N., Zarnowiecki D., Cho J., Wilson A., Bogomolova S., Villani A., Itsiopoulos C., Niyonsenga T., Blunden S., Meyer B. (2017). A Mediterranean-style dietary intervention supplemented with fish oil improves diet quality and mental health in people with depression: A randomized controlled trial (HELFIMED). Nutr. Neurosci..

[6] Psaltopoulou T., Sergentanis T.N., Panagiotakos D.B., Sergentanis I.N., Kosti R., Scarmeas N. (2013). Mediterranean diet, stroke, cognitive impairment, and depression: A meta-analysis. Ann. Neurol..

[7] Radd-Vagenas S., Duffy S.L., Naismith S.L., Brew B.J., Flood V.M., Fiatarone Singh M.A. (2018). Effect of the Mediterranean diet on cognition and brain morphology and function: A systematic review of randomized controlled trials. Am. J. Clin. Nutr..

[8] Ryan M.C., Itsiopoulos C., Thodis T., Ward G., Trost N., Hofferberth S., O’Dea K., Desmond P.V., Johnson N.A., Wilson A.M. (2013). The Mediterranean diet improves hepatic steatosis and insulin sensitivity in individuals with non-alcoholic fatty liver disease. J. Hepatol..

[9] Sofi F., Cesari F., Abbate R., Gensini G.F., Casini A. (2008). Adherence to Mediterranean diet and health status: Meta-analysis. BMJ.

[10] Sofi F., Macchi C., Abbate R., Gensini G.F., Casini A. (2014). Mediterranean diet and health status: An updated meta-analysis and a proposal for a literature-based adherence score. Public Health Nutr..

[11] Trichopoulou A., Costacou T., Bamia C., Trichopoulos D. (2003). Adherence to a Mediterranean diet and survival in a Greek population. N. Engl. J. Med..

[12] D’Alessandro A., De Pergola G. (2018). The Mediterranean Diet: Its definition and evaluation of a priori dietary indexes in primary cardiovascular prevention. Int. J. Food Sci. Nutr..

[13] Radd-Vagenas S., Kouris-Blazos A., Fiatarone Singh M., Flood V.M. (2017). Evolution of Mediterranean diets and cuisine: Concepts and definitions. Asia Pac. J. Clin. Nutr..

[14] Locke A., Schneiderhan J., Zick S.M. (2018). Diets for health: Goals and guidelines. Am. Fam. Phys..

[15] Martínez-González M.A., Sánchez-Tainta A., Corella D., Salas-Salvadó J., Ros E., Arós F., Gómez-Gracia E., Fiol M., Lamuela-Raventós R.M., Schröder H. (2014). A provegetarian food pattern and reduction in total mortality in the Prevención con Dieta Mediterránea (PREDIMED) study. Am. J. Clin. Nutr..

[16] Uribarri J., Woodruff S., Goodman S., Cai W., Chen X., Pyzik R., Yong A., Striker G.E., Vlassara H. (2010). Advanced glycation end products in foods and a practical guide to their reduction in the diet. J. Am. Diet. Assoc..

[17] D’Alessandro A., De Pergola G. (2015). Mediterranean diet and cardiovascular disease: A critical evaluation of a priori dietary indexes. Nutrients.

[18] Davis C., Bryan J., Hodgson J., Murphy K. (2015). Definition of the Mediterranean diet: A literature review. Nutrients.

[19] Hoffman R., Gerber M. (2013). Evaluating and adapting the Mediterranean diet for non-Mediterranean populations: A critical appraisal. Nutr. Rev..

[20] Kim H., Caulfield L.E., Rebholz C.M. (2018). Healthy plant-based diets are associated with lower risk of all-cause mortality in US adults. J. Nutr..

[21] Satija A., Bhupathiraju S.N., Rimm E.B., Spiegelman D., Chiuve S.E., Borgi L., Willett W.C., Manson J.E., Sun Q., Hu F.B. (2016). Plant-based dietary patterns and incidence of type 2 diabetes in US men and women: Results from three prospective cohort studies. PLoS Med..

[22] Radd-Vagenas S., Fiatarone Singh M.A., Inskip M., Mavros Y., Gates N., Wilson G.C., Jain N., Meiklejohn J., Brodaty H., Wen W. (2018). Reliability and validity of a Mediterranean diet and culinary index (MediCul) tool in an older population with mild cognitive impairment. Br. J. Nutr..

[23] Tognon G., Hebestreit A., Lanfer A., Moreno L.A., Pala V., Siani A., Tornaritis M., De Henauw S., Veidebaum T., Molnár D. (2013). Mediterranean diet, overweight and body composition in children from eight European countries: Cross-sectional and prospective results from the IDEFICS study. Nutr. Metab. Cardiovasc. Dis..

[24] Sotos-Prieto M., Santos-Beneit G., Bodega P., Pocock S., Mattei J., Penalvo J.L. (2015). Validation of a questionnaire to measure overall Mediterranean lifestyle habits for research application: The MEDiterranean LIFEstyle index (MEDLIFE). Nutr. Hosp..

[25] Trichopoulou A., Kouris-Blazos A., Wahlqvist M.L., Gnardellis C., Lagiou P., Polychronopoulos E., Vassilakou T., Lipworth L., Trichopoulos D. (1995). Diet and overall survival in elderly people. BMJ.

[26] Panagiotakos D.B., Milias G.A., Pitsavos C., Stefanadis C. (2006). MedDietScore: A computer program that evaluates the adherence to the Mediterranean dietary pattern and its relation to cardiovascular disease risk. Comput. Methods Programs Biomed..

[27] Rumawas M.E., Dwyer J.T., Mckeown N.M., Meigs J.B., Rogers G., Jacques P.F. (2009). The development of the Mediterranean-style dietary pattern score and its application to the American diet in the Framingham Offspring Cohort. J. Nutr..

[28] Schroder H., Fito M., Estruch R., Martinez-Gonzalez M.A., Corella D., Salas-Salvado J., Lamuela-Raventos R., Ros E., Salaverria I., Fiol M. (2011). A short screener is valid for assessing Mediterranean diet adherence among older Spanish men and women. J. Nutr..

[29] Cerwinske L.A., Rasmussen H.E., Lipson S., Volgman A.S., Tangney C.C. (2017). Evaluation of a dietary screener: The Mediterranean Eating Pattern for Americans tool. J. Hum. Nutr. Diet..

[30] Alberti-Fidanza A., Fidanza F. (2004). Mediterranean adequacy index of Italian diets. Public Health Nutr..

[31] Vitale M., Racca E., Izzo A., Giacco A., Parente E., Riccardi G., Giacco R. (2018). Adherence to the traditional Mediterranean diet in a population of South of Italy: Factors involved and proposal of an educational field-based survey tool. Int. J. Food Sci. Nutr..

[32] Cade J., Thompson R., Burley V., Warm D. (2002). Development, validation and utilisation of food-frequency questionnaires-A review. Public Health Nutr..

[33] Peat J. (2001). Health Science Research: A Handbook of Quantitative Methods.

[34] 45 and Up Study Collaborators (2008). Cohort Profile: The 45 and Up Study. Int. J. Epidemiol..

[35] Heffernan M., Andrews G., Fiatarone Singh M.A., Valenzuela M., Anstey K., Maeder A., McNeil J.J., Jorm L., Lautenschlager N., Sachdev P. (2018). Maintain Your Brain: Protocol of a 3-year randomised controlled trial of an individualised multi-modal eHealth intervention to prevent cognitive decline amongst community dwelling 55 to 77 year olds. J. Alzheimers Dis..

[36] Apple App Store Research Food Diary Xyris Software (Australia) Pty Ltd.. https://itunes.apple.com/au/app/research-food-diary/id1043440131.

[37] Ambrosini G.L., Hurworth M., Giglia R., Trapp G., Strauss P. (2018). Feasibility of a commercial smartphone application for dietary assessment in epidemiological research and comparison with 24-h dietary recalls. Nutr. J..

[38] Lieffers J.R.L., Hanning R.M. (2012). Dietary assessment and self-monitoring: With nutrition applications for mobile devices. Can. J. Diet. Pract. Res..

[39] Ingwersen L., Raper N., Anand J., Moshfegh A. (2004). Validation study shows importance of probing for forgotten foods during a dietary recall (abstract). J. Am. Diet. Assoc..

[40] NIH National Cancer Institute Automated Self-Administered 24-hour (ASA24) Dietary Assessment Tool. https://epi.grants.cancer.gov/asa24/respondent/methodology.html.

[41] Australian Bureau of Statistics (2014). 4364.0.55.007-Australian Health Survey: Nutrition First Results-Foods and Nutrients, 2011–2012.

[42] Martínez-González M.A., García-Arellano A., Toledo E., Salas-Salvado J., Buil-Cosiales P., Corella D., Covas M.I., Schröder H., Arós F., Gómez-Gracia E. (2012). A 14-item Mediterranean diet assessment tool and obesity indexes among high-risk subjects: The PREDIMED trial. PLoS ONE.

[43] Rosner B. (2015). Fundamentals of Biostatistics.

[44] Bland J.M., Altman D.G. (1986). Statistical methods for assessing agreement between two methods of clinical measurement. Lancet.

[45] Landis J.R., Koch G.G. (1977). The measurement of observer agreement for categorical data. Biometrics.

[46] National Health and Medical Research Council (2013). Clinical Practice Guidelines for the Management of Overweight and Obesity in Adults, Adolescents and Children in Australia.

[47] Papadaki A., Johnson L., Toumpakari Z., England C., Rai M., Toms S., Penfold C., Zazpe I., Martínez-Gonzalez M.A., Feder G. (2018). Validation of the English version of the 14-item Mediterranean Diet Adherence Screener of the PREDIMED study, in people at high cardiovascular risk in the UK. Nutrients.

[48] Schröder H., Marrugat J., Vila J., Covas M.I., Elosua R. (2004). Adherence to the traditional Mediterranean diet is inversely associated with body mass index and obesity in a Spanish population. J. Nutr..

[49] Toledo E., Salas-Salvadó J., Donat-Vargas C., Buil-Cosiales P., Estruch R., Ros E., Corella D., Fitó M., Hu F.B., Arós F. (2015). Mediterranean diet and invasive breast cancer risk among women at high cardiovascular risk in the PREDIMED trial: Randomized clinical trial. JAMA. Intern. Med..

[50] Valls-Pedret C., Sala-Vila A., Serra-Mir M., Corella D., de la Torre R., Martínez-González M.Á., Martínez-Lapiscina E.H., Fitó M., Pérez-Heras A., Salas-Salvadó J. (2015). Mediterranean diet and age-related cognitive decline: A randomized clinical trial. JAMA. Intern. Med..

[51] Australian Bureau of Statistics National Health Survey: First Results, 2014-15-Australia, ‘Table 8.3 Body Mass Index, Waist Circumference, Height and Weight’, Time Series Spreadsheet, Cat. No. 4364.0.55.001. http://www.abs.gov.au/AUSSTATS/abs@.nsf/DetailsPage/4364.0.55.0012014-15?OpenDocument.

[52] Australian Bureau of Statistics National Health Survey: First Results, 2014-15-Australia, ‘Table 3.3 Long-Term Health Conditions’, Time Series Spreadsheet, Cat. No. 4364.0.55.0013. http://www.abs.gov.au/AUSSTATS/abs@.nsf/DetailsPage/4364.0.55.0012014-15?OpenDocument.

[53] Thompson F.E., Subar A.F., Coulston A.M., Boushey C.J., Ferruzzi M.G., Delahanty L.M. (2017). Chapter 1-Dietary Assessment Methodology. Nutrition in the Prevention and Treatment of Disease.

[54] Ambrosini G.L., Van Roosbroeck S.A.H., Mackerras D., Fritschi L., De Klerk N.H., Musk A.W. (2003). The reliability of ten-year dietary recall: Implications for cancer research. J. Nutr..

