# Supplementary material for: Validity of the Mediterranean Diet and Culinary Index (MediCul) for Online Assessment of Adherence to the ‘Traditional’ Diet and Aspects of Cuisine in Older Adults
Source: Nutrients. 2018 Dec 4;10(12):1913. doi: 10.3390/nu10121913 (PMC6315571; doi:10.3390/nu10121913)
Supplement: Supplementary file 1 [file nutrients-10-01913-s001.zip › nutrients-388368/Table S1-How to derive MEDAS score from MediCul.pdf]

Table S1: How to derive a MEDAS score from MediCul

Aim: To derive a Mediterranean Diet Adherence Screener (MEDAS) score out of 14 from the responses provided to the 50-item Mediterranean Diet and Culinary Index (MediCul) tool.

Instructions:

1. Check the participant responses to the relevant MediCul question numbers listed in column three below.
2. Convert these responses to the relevant frequency for scoring, where required*.
3. Follow the instructions in columns four and five below to score each MEDAS question either 1 or 0 (zero) points; if a response cannot be awarded 1 point, it is automatically awarded a 0 (zero) point as there are no intermediate points.
4. Add up the points for the 14 MEDAS questions to provide the total MEDAS score.

Note – some MEDAS questions used within MediCul have been optimised for the English language.

**1. MEDAS question [1]**	**2. MEDAS criteria for 1 point**	**3. Relevant MediCul Question/s [2]**	**4. MediCul response category**	**5. MEDAS scoring instructions**
1. Do you use olive oil as main culinary fat?	Yes	Q. 29	This MediCul question is only used to score for MEDAS, hence no adjustment is required	This MediCul question is only used to score for MEDAS, hence no adjustment is required
2. How much olive oil do you consume in a given day (including oil used for frying, salads, out-of-house meals, etc.)?	≥ 4 TBSP/d	Q. 28	First check that response to Q. 28 is given per day or convert to per day*	Apply MEDAS criteria (column 2) and score

1. MEDAS question [1]	2. MEDAS criteria for 1 point	3. Relevant MediCul Question/s [2]	4. MediCul response category	5. MEDAS scoring instructions
3. How many vegetable servings do you consume per day? [1 serving: 200 g (consider side dishes as half a serving)]	≥ 2 (≥ 1 portion raw or as a salad)	Q. 1	First check that response to Q. 1 is given per day or convert to per day*	Take total MediCul serves/d and multiply by 75 g then divide by 200 g;<br><br>Apply MEDAS criteria (column 2) and score
4. How many fruit units (including natural fruit juices) do you consume per day?	≥ 3 serve/d	Q. 12 and Q. 34	First check that responses given in Q. 12 and Q. 34 are expressed as per day or convert to per day*;<br>Finally, add together responses from Q. 12 and Q. 34	Apply MEDAS criteria (column 2) and score
5. How many servings of red meat, hamburger or meat products (ham, sausage, etc.) do you consume per day?	< 1 serve/d	Q. 13 and Q. 14	First check that responses given in Q. 13 and Q. 14 are expressed as per day or convert to per day*;<br>Add together responses for Q. 13 and 14	Apply MEDAS criteria (column 2) and score
6. How many servings of butter, margarine, or cream do you consume per day? (1 serving: 12 g)	< 1 serve/d	Q. 26 and Q. 27	First check that responses given in Q. 26 and Q. 27 are expressed as per day or convert to per day*;<br>Add together daily serves for Q. 26 and Q. 27	Divide total for Q. 26 & Q. 27 by 2 to correct for MEDAS serve size;<br><br>Apply MEDAS criteria (column 2) and score
7. How many sweet/carbonated beverages do you drink per day?	< 1/d	Q. 33	First check that response given in Q. 33 is expressed as per day or convert to per day*	Apply MEDAS criteria (column 2) and score

1. MEDAS question [1]	2. MEDAS criteria for 1 point	3. Relevant MediCul Question/s [2]	4. MediCul response category	5. MEDAS scoring instructions
8. How much wine do you drink per week?	≥ 7 glasses/ wk	Q. 41c	First check that response given in Q. 41c is expressed as per week or convert to per week*	Apply MEDAS criteria (column 2) and score
9. How many servings of legumes do you consume per week? (1 serving: 150 g)	≥ 3/wk	Q. 19	First check that response given in Q. 19 is expressed as per week or convert to per week*	Apply MEDAS criteria (column 2) and score
10. How many servings of fish or shellfish do you consume per week? (1 serving: 100-150 g of fish or 4-5 units or 200 g shellfish)	≥ 3/wk	Q. 16	First check that response given in Q. 16 is expressed as per week or convert to per week*	Apply MEDAS criteria (column 2) and score
11. How many times per week do you consume commercial sweets or pastries (not homemade), such as cakes, cookies, biscuits or custard?	< 3/wk	Q. 31, Q. 32a	ASSUMPTION: serves/wk for cakes/biscuits = times/wk;<br>First check that response given in Q. 31 is expressed as per week or convert to per week*;<br>Add Q. 31 response + Q.32a response (custard only)	Apply MEDAS criteria (column 2) and score
12. How many servings of nuts (including peanuts) do you consume per week? (1 serving: 30 g)	≥ 3/wk	Q. 24	First check that response given in Q. 24 is expressed as per week or convert to per week*	Apply MEDAS criteria (column 2) and score

1. MEDAS question [1]	2. MEDAS criteria for 1 point	3. Relevant MediCul Question/s [2]	4. MediCul response category	5. MEDAS scoring instructions
13. Do you preferentially consume chicken, turkey or rabbit meat instead of veal, pork, hamburger or sausage?	Yes	Q. 17	Assume Yes = if ‘chicken, turkey or rabbit’ OR ‘I don’t eat chicken or meat’ options ticked;<br>Assume No = if ‘beef, pork, hamburgers or sausages’ ticked	Apply MEDAS criteria (column 2) and score
14. How many times per week do you consume vegetables, pasta, rice or other dishes seasoned with sofrito (sauce made with tomato and onion, leek or garlic and simmered with olive oil)?	≥ 2/wk	Q. 6	First check that response given in Q. 6 is expressed as per week or convert to per week*	Apply MEDAS criteria (column 2) and score

Abbreviations: Q, question; TBSP, tablespoons; d, day; g, grams; wk, week.
*As the MediCul questions may include daily, weekly and monthly response options, some conversions are required to ensure responses relate to the same time period as in the MEDAS questions:

- To convert responses given for serves/frequency per month to serves/frequency per week: divide by 30 then multiply by 7.
- To convert responses given for serves/frequency per week to serves/frequency per day: divide by 7.
- To convert responses given for serves/frequency per month to serves/frequency per day: divide by 30.

References for MEDAS and MediCul tools and their scoring

1. Schroder, H.; Fito, M.; Estruch, R.; Martinez-Gonzalez, M.A.; Corella, D.; Salas-Salvado, J.; Lamuela-Raventos, R.; Ros, E.; Salaverria, I.; Fiol, M., et al. A short screener is valid for assessing Mediterranean diet adherence among older Spanish men and women. *J. Nutr*. **2011**, *141*, 1140-1145, doi:10.3945/jn.110.135566.
2. Radd-Vagenas, S.; Fiatarone Singh, M.A.; Inskip, M.; Mavros, Y.; Gates, N.; Wilson, G.C.; Jain, N.; Meiklejohn, J.; Brodaty, H.; Wen, W., et al. Reliability and validity of a Mediterranean diet and culinary index (MediCul) tool in an older population with mild cognitive impairment. *Br. J. Nutr*. **2018**, *120*, 1189-1200, doi:10.1017/S0007114518002428.
