# Supplementary material for: Validity of the Mediterranean Diet and Culinary Index (MediCul) for Online Assessment of Adherence to the ‘Traditional’ Diet and Aspects of Cuisine in Older Adults
Source: Nutrients. 2018 Dec 4;10(12):1913. doi: 10.3390/nu10121913 (PMC6315571; doi:10.3390/nu10121913)
Supplement: Supplementary file 1 [file nutrients-10-01913-s001.zip › nutrients-388368/Table S2-Kappa statistics for percent agreement within the same category of 17 MediCul groups, between survey A and survey B.pdf]

Table S2: Kappa statistics for percent agreement within the same category of 17 MediCul groups, between survey A and survey B

MediCul group	% Agreement within same category	Kappa value	Level of agreement [1]
1. Olive oil	59.5%	0.47 (p<0.0001)	Moderate
2. Vegetables	32.4%	0.21 (p<0.0001)	Fair
3. Fruit	81.1%	0.63 (p<0.0001)	Substantial
4. Nuts	64.9%	0.50 (p<0.0001)	Moderate
5. Wholegrains	93.2%	0.77 (p<0.0001)	Substantial
6. Legumes	67.6%	0.49 (p<0.0001)	Moderate
7. Fish/shellfish	82.4%	0.69 (p<0.0001)	Substantial
8. Eggs	90.5%	0.74 (p<0.0001)	Substantial
9. Dairy products	86.5%	0.63 (p<0.0001)	Substantial
10. White meat preference	70.3%	0.50 (p<0.0001)	Moderate
11. Red/processed meat	63.5%	0.53 (p<0.0001)	Moderate
12. Sweets & sugary drinks	59.5%	0.51 (p<0.0001)	Moderate
13. Takeaway	82.4%	0.64 (p<0.0001)	Substantial
14. Water	78.4%	0.71 (p<0.0001)	Substantial
15. Alcohol	66.2%	0.58 (p<0.0001)	Moderate
16. Coffee	100.0%	1.00 (p<0.0001)	Almost perfect
17. Cuisine	39.2%	0.23 (p<0.0001)	Fair

Abbreviations: survey A, first administration of MediCul tool; survey B, second administration of MediCul tool.

Reference for interpretation of Kappa values

1. Landis, J.R.; Koch, G.G. The measurement of observer agreement for categorical data. *Biometrics* **1977**, *33*, 159-174.
